# Supplementary material for: The Evolution and Characterization of the RNA Interference Pathways in Lophotrochozoa
Source: Genome Biol Evol. 2024 May 7;16(5):evae098. doi: 10.1093/gbe/evae098 (PMC11114477; doi:10.1093/gbe/evae098)
Supplement: evae098_Supplementary_Data [file evae098_supplementary_data.zip › Supplementary_materials.zip!/selected_miRNAs/NoGe_selected_miRNAs/known_miRNA_families_pdf_files/NMRB01000161.1_Notospermus_geniculatus_strain_Ushimado_scaffold161,_whole_genome_shotgun_sequence_6107.pdf]

Provisional ID	:	NMRB01000161.1_Notospermus_geniculatus_strain_Ushimado_scaffold161,_whole_genome_shotgun_sequence_6107
Score total	:	4741.1
Score for star read(s)	:	3.9
Score for read counts	:	4732.8
Score for mfe	:	1.3
Score for randfold	:	0
Score for cons. seed	:	3
Total read count	:	9295
Mature read count	:	9089
Loop read count	:	0
Star read count	:	206

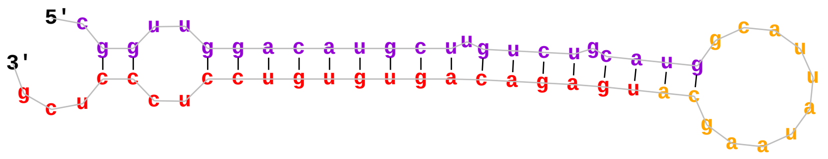

Genome coordinates: NMRB01000161.1 422305-422363

pre-miRNA sequence:
CGGUUGGACAUGCUUGUCUGCAUGCGGUUGGACAUGCUUGUCUGCAUGGCAUUAUAAGCAUGAGACAGUGUGUCCUCCCUCG

Mature sequence: UGAGACAGUGUGUCCUCCCUCG

Aligned mature sequence of the *N. geniculatus* pre-miRNA with the top 5 hits from the BLAST search against the pre-miRNA mirGeneDB set:

```
                            ********************
Nge_Mir-1994-P1             ugagacagugugucccucccucg
Lan-Mir-1994-P3a_3p         ugagacagugugucccucccucg
Lan-Mir-1994-P3b_3p         ugagacagugugucccucccucg
Lgi-Mir-1994-P2_3p          ugagacagugugucccucccuca
Lgi-Mir-1994-P1_3p          ugagacagugugucccucccuug
Cgi-Mir-1994-P1_3p          ugagacagugugucccucccuug
```
